# Telemedicine interventions for hypertension management in low- and middle-income countries: A scoping review

**DOI:** 10.1371/journal.pone.0254222

**Published:** 2021-07-09

**Authors:** Michael Hoffer-Hawlik, Andrew Moran, Lillian Zerihun, John Usseglio, Jennifer Cohn, Reena Gupta

**Affiliations:** 1 Columbia University Vagelos College of Physicians and Surgeons, New York, New York, United States of America; 2 Resolve to Save Lives, an initiative of Vital Strategies, New York, New York, United States of America; 3 Division of Infectious Diseases, Department of Medicine, University of Pennsylvania, Philadelphia, Pennsylvania, United States of America; 4 Division of General Internal Medicine, Department of Medicine, University of California San Francisco, San Francisco, California, United States of America; National School of Public Health, Institute of Health Carlos III, SPAIN

## Abstract

Hypertension remains the leading cause of cardiovascular disease worldwide and disproportionately impacts patients living in low- and middle-income countries (LMICs). Telemedicine offers a potential solution for improving access to health care for vulnerable patients in LMICs.

**Objectives:**

The purpose of this scoping review was to summarize the evidence for telemedicine interventions for blood pressure management in LMICs and assess the relationships between the telemedicine intervention characteristics and clinical outcomes.

**Design:**

Published studies were identified from the following databases (from their inception to May 2020): PubMed, Scopus, and Embase. Search terms related to “Low and Middle Income Countries,” “Telemedicine,” and “Hypertension” were used, and clinical outcomes were extracted from the screened articles.

**Results:**

Our search resulted in 530 unique articles, and 14 studies were included in this review. Five studies assessed telemedicine interventions for patient-provider behavioral counseling, four assessed patient-provider medical management, and five assessed provider-provider consultation technologies. Out of fourteen individual studies, eleven demonstrated a significant improvement in systolic or diastolic blood pressure in the intervention group. Of the eight studies that reported difference-in-differences changes in systolic blood pressure, between-arm differences ranged from 13.2 mmHg to 0.4 mmHg.

**Conclusions:**

The majority of the studies in this review demonstrated a significant reduction in blood pressure with use of the telemedicine intervention, though the magnitude of benefit was not consistently large. Limitations of the studies included small sample sizes, short duration, and intervention heterogeneity. Current evidence suggests that telemedicine may provide a promising approach to increase access to care and improve outcomes for hypertension in LMICs, especially during events that limit access to in-person care, such as the COVID-19 pandemic. However, high-quality clinical trials of sufficient size and duration are needed to establish the impact and role of telemedicine in hypertension care. The protocol for this review was not registered.

## Introduction

According to a recent World Health Organization report on non-communicable diseases (NCDs), 71% of global deaths in 2016 were attributed to NCDs with cardiovascular diseases accounting for 44% of NCD deaths [1]. By 2030, the global cost of cardiovascular disease is projected to reach US$20 trillion [2]. Hypertension is the leading cause of death worldwide and accounts for over 10 million deaths annually. It is currently estimated that 1 in 3 adults are affected by hypertension in low- and middle-income countries (LMICs). and approximately 3 out of 4 NCD deaths occur in LMICs [1,3].

The significant health and economic burden of hypertension in LMICs is further complicated by lower proportions of treatment and control compared to high-income countries [3,4]. Less than 15% of people with hypertension worldwide have controlled blood pressure [3]. In addition to the ongoing challenges related to hypertension management in LMICs, the COVID-19 pandemic introduced a unique set of barriers for patients to access chronic disease care. Restrictions on travel limit the ability for patients to access care and medications in person. Many LMICs possess overcrowded health systems which face challenges in effectively introducing physical distancing and infection control measures [5]. Further, patients with cardiovascular disease are at higher risk for severe COVID-19 infection, and these patients may risk exposure to COVID-19 while seeking care [6].

Telemedicine, the remote diagnosis and treatment of patients by means of telecommunications technology, offers a promising approach to improving the accessibility and affordability of care for hypertension patients in LMICs during the era of COVID-19 and beyond the pandemic [7,8]. Telemedicine has the potential to reduce barriers to primary healthcare in LMICs, including transportation burden, overcrowded facilities, and healthcare workforce shortages [9]. While several studies have investigated the scope of telehealth and mobile services for NCD management in LMICs, many of these reviews assessed technology platforms for one-way communication such as unidirectional text messaging or automated patient reminders [10–12]. To our knowledge, there are few studies specifically evaluating telemedicine applications involving direct communication between healthcare providers or between providers and patients. We define telemedicine as direct interactive electronic communication between the patient and the provider or between multiple providers in either synchronous or asynchronous settings for provision of health care services or consultation [13]. Through telemedicine, remote and vulnerable patient populations may obtain instant access to tailored care through remote providers. Literature reviews addressing telemedicine interventions for blood pressure management in LMICs are limited [11,14].

The aims of this study were to 1) systematically review the available evidence for telemedicine interventions for hypertension and blood pressure management in LMICs and 2) assess the relationships between the telemedicine intervention characteristics and clinical outcomes. Based on the aims of this study, a scoping review was performed given the unestablished nature of the relevant body of literature and the high degree of study heterogeneity. Understanding the effectiveness of telemedicine services in LMICs will inform successful approaches to maintaining essential health services for patients with hypertension throughout the COVID-19 pandemic as well future implementation in LMICs to reduce disparities in hypertension-related health outcomes.

## Methods

### Protocol and registration

MHH, LZ, JU, AM, and RG developed the protocol for the scoping review and database search strategy. The protocol for this review was not registered. This scoping review was reported in accordance with the Preferred Reporting Items for Systematic Reviews and Meta-analyses extension for Scoping Reviews (PRISMA-ScR) (S1 Data) [15]. The scoping review methodology was conducted in accordance with the framework proposed by Arksey and O’Malley [16].

### Information sources and search strategy

Published studies in PubMed, Scopus, and Embase databases were searched using key terms and Boolean operators related to 1) “Low and Middle Income Countries,” 2) “Telemedicine,” and 3) “Hypertension” (S1 Table). The list of LMICs was obtained from the World Bank Country and Lending Groups list for low-income and lower-middle income economies [17]. The search included studies published since the inception of the databases. The final search was performed in May 21, 2020.

### Eligibility criteria

Articles were included in the review if the studies met the following criteria: (1) evaluated changes in blood pressure outcomes resulting from the study intervention; (2) assessed bi-directional telemedicine interventions that include direct interactions between patients and health professionals or between health professionals to inform the evaluation, diagnosis, treatment, or prevention of hypertension disease among the intervention population; (3) conducted in LMICs; (4) reported health-related outcomes among participants as a result of the intervention; (5) peer-reviewed; and (6) published in English language. Articles were excluded from the review if they failed to meet these inclusion criteria or if the study assessed interventions related to hypertension in pregnancy or secondary complications of hypertension.

### Screening and selection process

All identified studies were imported into EndNote X8 reference management software and duplicates were deleted. The articles were then screened using Covidence, a systematic review software [18]. Two authors (MHH and LZ) independently conducted the screening process in a blinded manner. Any conflicts that appeared during the independent evaluations were discussed, and if any disagreement persisted, a third investigator (RG) reviewed the articles and the majority opinion was applied.

### Data charting process

The data was extracted independently by MHH and LZ and was entered into a standardized Microsoft Excel form developed by MHH, AM, LZ, and RG. Both abstractors confirmed that the data was entered accurately. Discrepancies were resolved through discussion, and if any disagreement persisted, a third abstractor (RG) was consulted. Data was extracted as reported in the studies, and study authors were not contacted when article information was unclear or not reported.

### Data items

For each of the included full-text studies, the following data was extracted: author, publication year, country, types of non-communicable disease(s) among the study participants, inclusion criteria, sample size, study design and follow-up duration, description of the telemedicine component of the study intervention, and outcomes reported by the studies related to systolic blood pressure, diastolic blood pressure, blood pressure control, HgA1c, blood sugar, and cholesterol levels. To assess the relationship between telemedicine intervention characteristics and outcomes, the included studies were categorized into one of three modes of communication: telephone, video chat, and electronic messaging. Within each category of communication mode, studies were also categorized into one of three intervention types: patient-provider behavioral counseling, patient-provider medical management, and provider-provider consultation.

### Synthesis of results

The charted data for each study was presented in narrative tabular formats. Narrative descriptions were provided for the inclusion criteria, study design and follow-up, and the telemedicine component for each study intervention. Numerical descriptions were also provided for study sample size and reported outcomes with level of significance, if available. Blood pressure measures included in the narrative table were converted to mmHg if presented in an alternative measurement within the original study report. Blood pressure outcomes for each study were also presented using color-based visual depictions in a tabular format to display level of significance across mode of communication and intervention type by study. Meta-analytic techniques were not performed due to heterogeneity of the studies concerning type of telemedicine intervention, study participants, and reported outcomes, thereby precluding meaningful analysis of pooled data.

## Results

### Literature search results

**Fig 1:**
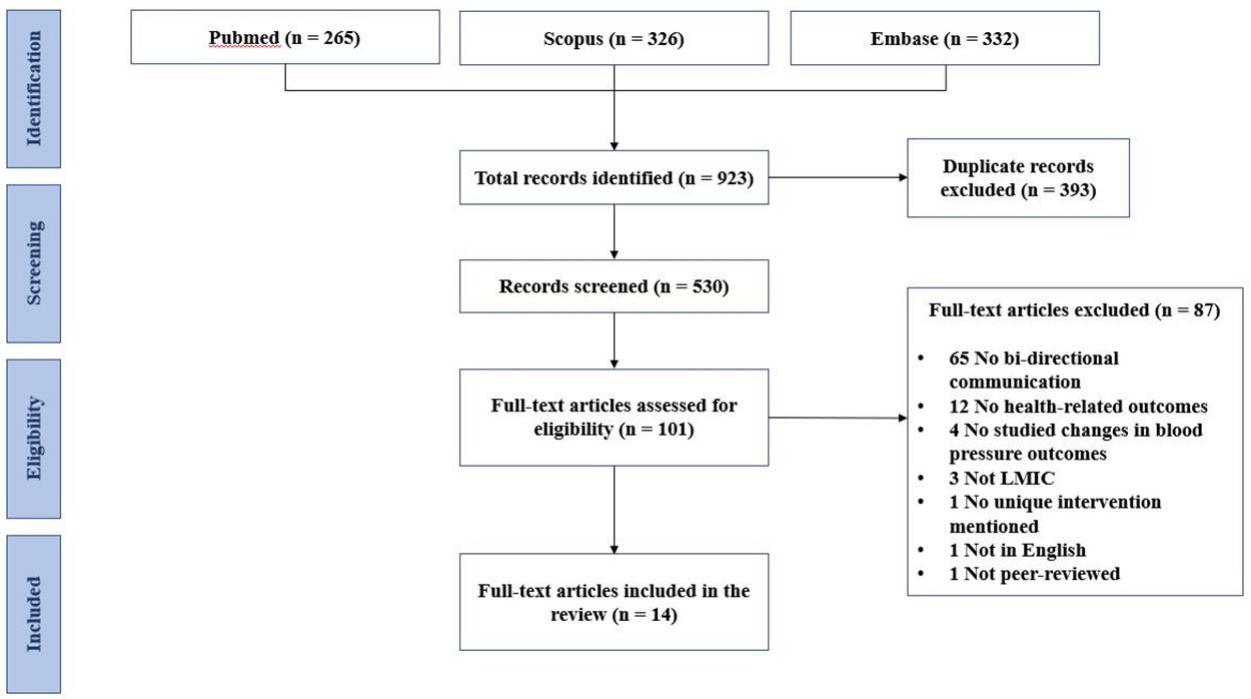
Flow-diagram of search process.

Our search identified a total of 923 articles through PubMed (n = 265), Scopus (n = 326), and Embase (n = 332). 393 duplicate articles were removed, yielding a total of 530 unique articles for title and abstract screening. 429 articles did not meet eligibility criteria and were excluded from consideration. Full-text articles of 101 studies were retrieved and analyzed. Out of the 101 full-text articles, 87 articles were excluded, resulting in 14 studies that met all inclusion and exclusion criteria (Fig 1).

### Characteristics of included studies

#### Study location and period

India was the most common location of the included studies (n = 4) (Table 1) [19–22]. There were two studies conducted in China [23,24], and one study each from Turkey [25], Cameroon [26], Vietnam [27], Nigeria [28], Bangladesh [29], Indonesia [30], and Brazil [31]. One study was conducted in Argentina, Guatemala, and Peru [32]. The fourteen studies were conducted between 2013 and 2020.

**Table 1:** Overview of telemedicine interventions for hypertension management in LMICs.

Study (country)	Non-communicable disease(s)	Inclusion criteria	Sample size	Study design and follow-up	Description of telemedicine component of intervention	Outcomes (reported as difference in differences unless otherwise specified)
Dandge et al., 2019 (India) [19]	Hypertension, diabetes	Adults aged 20 years and above Located in two randomly selected villages from Medchal	1,835 (413 with hypertension, 189 with diabetes)	Cross-sectional with pre- and post-test analysis; follow-up after 24 months	Video Skype call between the participant and the physician using mHealth application coordinated by non-physician health workers.	54% achieved BP control status SBP ↓ 6.5 mmHg (P<0.001) and DBP ↓ 5.9 mmHg (P<0.001) among participants with a past medical history of hypertension SBP ↓ 18.1 mm Hg (P<0.001) and DBP ↓ 15.3 mm Hg (P<0.001) among participants with newly detected hypertension
Kingue et al., 2013 (Cameroon) [26]	Hypertension	Adults aged 15 years and above SBP≥140 mmHg or DBP ≥90 mmHg SBP≥130 mmHg or DBP≥80 mmHg for those with diabetes or nephropathy At least 12 months of continuous residence in the study areas prior to the study	268 (intervention = 165, control = 103)	Prospective interventional study; follow-up after 24 weeks	Staff located at remote treatment centers could immediately consult with a hospital telemedicine center via mobile phone for clinical decision making.	SBP ↓ (P = 0.01) DBP ↓ (P = 0.0002) 50% of intervention group and 39.1% of control group achieved target BP levels (P = 0.04) among participants with stage 3 hypertension 65.2% of intervention group and 70% of control group achieved target BP levels (P = 0.20) among participants with hypertension stage I-II
Kanadli et al., 2016 (Turkey) [25]	Diabetes	Diagnosed with diabetes in the previous year	88 (intervention = 44, control = 44)	Randomized controlled trial; follow-up after 3 months	Received telephone follow-up weekly in the first month and biweekly in the second and third months. Metabolic control values reviewed, and continued education provided during calls.	SBP ↓ 5.91 mmHg (P = 0.011) DBP ↓ 2.72 mmHg (P = 0.21)
Lee et al., 2018 (Vietnam) [27]	Hypertension, diabetes	Overseas Korean patients with hypertension, diabetes mellitus, or both	234 total; 36 received two or more telehealth counselling sessions (intervention = 10, control = 26)	Cross-sectional with pre- and post-test analysis; follow-up after 3 months	Received telehealth counseling from a Korean doctor through a telehealth network system.	SBP ↓ 6.4 mmHg and DBP ↓ 4.4 mmHg for all patients SBP ↓ 13.2 mmHg (P = 0.0076) between monitoring and non-monitoring group DBP ↓ 1.7 mmHg (P = 0.67) between monitoring and non-monitoring group
Li et al., 2019 (China) [23]	Cardiovascular disease	Adults aged 45–70 years old Able to use WeChat Lived in Yuexiu District for at least six months Reported a definite diagnosis of hypertension while taking or having ever taken an antihypertensive drug	462 (intervention = 186, control = 276)	Randomized controlled trial; follow-up after 6 months	Private chats between participants and researchers via WeChat. Researchers consulted with community physicians for hypertension management plans via WeChat.	SBP ↓ 6.9 mmHg (P = 0.002) DBP ↓ 3.1 mmHg (P = 0.016) Hypertension control ↑ 22.7% (adjusted odds ratio: 5.0 (2.3, 11.3); P < 0.001) in the intervention group
Liu et al., 2015 (China) [24]	Cardiovascular disease	Adults aged 45–75 years without known cardiovascular disease	589 (intervention = 238, control = 351)	Randomized controlled trial; follow-up after 12 months	Received phone calls ranging from once per week to twice per month depending on 10-year risk of CVD. Calls focused on providing guidance related to modifiable risk factors and healthy lifestyle.	SBP ↓ 12.45 mmHg (P<0.05) DBP ↓ 12.23 mmHg (P<0.01)
Nelissen et al., 2018 (Nigeria) [28]	Hypertension	Adults aged 18 years and above New or previous hypertension diagnosis	336	Cross-sectional with pre- and post-test analysis; follow-up after 6 months	Pharmacists communicated with the cardiologists via an mHealth app for remote patient management.	SBP ↓ 9.9 mmHg (P<0.05) DBP ↓ 5.4 mmHg (P<0.05) BP on target ↑ 32% (P < 0.001)
Nohara et al., 2015 (Bangladesh) [29]	Hypertension, diabetes	Adults from rural and urban areas in Bangladesh	16,741 (2,361 participated in both health checkups)	Cross-sectional with pre- and post-test analysis; follow-up after 12 months	Patients classified as either "affected" or "emergent" were provided telemedicine consultations via Skype with a medical call center.	Identified 32.4% of subjects as affected and 54.1% of subjects as caution required Mean SBP ↓ 5 mmHg for all participants after second health checkup (P<0.001)
Patel et al., 2019 (Indonesia) [30]	Cardiovascular disease	Adults aged 40 years and older Living in one of eight intervention or control villages	22,635 (intervention = 11 647, control = 10 988)	Randomized controlled trial; follow-up after 11.5 (intervention initiation to end of follow up), 12.6 months (control)	Physicians and nurses received tailored decision support for treatment plans via a mobile application Treatment plans subsequently sent to community health workers.	SBP ↓ 8.3 mmHg (P<0.001) DBP ↓ 3.6 mmHg (P<0.001)
Patnaik et al., 2014 (India) [20]	Diabetes mellitus, coronary heart disease	Adults aged 30 years and above Treated for diabetes for at least 3 months	100 (intervention = 50, control = 50)	Randomized controlled trial; follow-up after 3 months	Participants contacted every 3 weeks for 3 months by telephone by the investigator, Asked about lifestyle changes and provided counseling.	No significant change in hypertension classification between intervention and control groups
Rubinstein et al., 2016 (Argentina, Guatemala, Peru) [32]	Hypertension	Adults 30–60 years old who owned mobile phones SBP and DBP in the prehypertension range (between 120 and 139 mmHg and between 80 and 89 mm Hg, respectively) Not receiving medication for hypertension	637 (intervention = 316, control = 321)	Randomized controlled trial; follow-up after 12 months	Monthly phone calls using motivational interview techniques. Discussed lifestyle modifications (e.g., reduction of sodium intake, promotion of physical activity).	SBP ↓ 1.13 mmHg (P = 0.31) at 6 months and ↓ 0.37 mmHg (P = 0.43) at 12 months DBP ↓ 0.45 mmHg (P = 0.44) at 6 months and ↑ 0.01 mmHg (P = 0.99) at 12 months
Ruschel et al., 2020 (Brazil) [31]	Coronary artery disease	Adults aged 18 years or older Diagnosed with coronary artery disease and class I or II angina meeting discharge criteria No cardiovascular event or decompensated clinical condition in past year	271 (intervention = 135, control = 136)	Randomized controlled trial; follow-up after 12 months	Patients were followed-up after discharge from a specialized outpatient clinical to a primary care unit with clinical support from a cardiologist available for telemedicine consults. Patients were interviewed via telephone.	BP control ↑ 1.10% (P>0.05)
Sharma et al., 2017 (India) [21]	Non-communicable diseases (NCD)	Adults aged 18 to 64 years who had been living in Barwala for at least 6 months	400 (intervention = 200, control = 200)	Cross-sectional with pre- and post-test analysis; follow-up after 8 months	Patients received telephone calls once a month and discussed behavioral modification of NCD risk factors and any questions with the researchers.	SBP ↓ 1.3 mmHg in intervention group (P<0.001) and ↑ 0.3 mmHg in control group (P = 0.08) DBP ↑ 0.3 mmHg in intervention group (P = 0.59) and ↑ 0.1 mmHg in control group (P = 0.47)
Vitale et al., 2015 (India) [22]	Diabetes	Adults between 25 and 80 years old Diagnosed with diabetes more than a year previously	175 (intervention = 100, control = 75)	Cross-sectional with pre- and post-test analysis; follow-up not reported	Patients provided with telephone appointments with a multi-disciplinary team of diabetologists, diabetes educators, dietitians, pharmacists, and psychologists.	SBP ↓ 4.1 mmHg (P = 0.20) DBP ↓ 4.1 mmHg (P = 0.016)

BP, blood pressure; SBP, systolic blood pressure; DBP, diastolic blood pressure.

#### Participant characteristics and study design

For the studies included in this review, the sample size ranged from 88 to 22,635 participants. There were seven randomized controlled trials [20,23–25,30–32], six pre- and post-test studies [19,21,22,27–29], and one prospective interventional study [26]. Of the 14 studies, eleven included participants with either hypertension or other cardiovascular disease. Three studies included patients with both diabetes and hypertension [19,27,29], one study included patients with diabetes and coronary artery disease [20], and two studies recruited patients with diabetes [22,25]. One study did not recruit patients with any specific NCD [21].

#### Categorizations of the interventions

The majority of the studies included in the review (n = 7) utilized telephone communication [20–22,24–26,32], four studies incorporated video chat applications [19,27,29,31], and three studies used electronic messaging platforms (Table 2) [23,28,30]. Five studies evaluated patient to provider telemedicine interventions to encourage behavioral risk factor modification such as bi-weekly telephone reminders to limit alcohol consumption and increase physical activity levels [20,21,24,25,32]. Five studies enabled provider to provider consultation to inform treatment decision-making, including technology platforms that allowed for asynchronous treatment consultations between community health workers and physicians and nurses [23,26,28,30,31]. Four studies allowed patients to directly engage with providers via telemedicine for direct medical management, such as Skype medicine consultation video calls between the physician and study participants [19,22,27,29]. In addition to telemedicine communication, there were a significant number of studies (n = 12) that also incorporated non-telemedicine mobile health components as part of the research intervention (S2 Table).

**Table 2:** Categorizations of telemedicine interventions.

Study	Mode of Communication	Intervention Type	Timing	Control Group
Dandge et al.	Video chat	Patient-provider medical management	Not reported	--
Kanadli et al.	Telephone	Patient-provider behavioral counseling	Weekly for first month and bi-monthly afterwards	Routine treatment
Kingue et al.	Telephone	Provider-provider consultation	Immediately upon initiating consult	Routine treatment
Lee et al.	Video chat	Patient-provider medical management	Not reported	Routine treatment
Li et al.	Electronic messaging	Provider-provider consultation	Not reported	Health lectures and one follow-up appointment every three months
Liu et al.	Telephone	Patient-provider behavioral counseling	Weekly to bi-monthly depending on CVD risk	Annual medical exam
Nelissen et al.	Electronic messaging	Provider-provider consultation	Immediately upon initiating consult	--
Nohara et al.	Video chat	Patient-provider medical management	Not reported	--
Patel et al.	Electronic messaging	Provider-provider consultation	Not reported	Routine treatment
Patnaik et al.	Telephone	Patient-provider behavioral counseling	Every 3 weeks for first 3 months	Printed educational materials
Rubinstein et al.	Telephone	Patient-provider behavioral counseling	Monthly	Routine treatment
Ruschel et al.	Video chat	Provider-provider consultation	Not reported	Routine treatment
Sharma et al.	Telephone	Patient-provider behavioral counseling	Monthly	Routine treatment
Vitale et al.	Telephone	Patient-provider medical management	Not reported	Routine treatment

### Hypertension outcomes of the telemedicine interventions

There were a total of three relevant clinical blood pressure outcomes compared either within the intervention arm (“pre-post” comparison) or between the intervention and usual care arms (“difference-in-differences” comparison) across the fourteen included studies: systolic blood pressure change, diastolic blood pressure change, or change in the proportion of participants who achieved target blood pressure levels (Table 3). Blood pressure thresholds used to define hypertension control varied among the studies, which reference different international hypertension guidelines in defining control. Out of the fourteen included studies, eleven showed a significant improvement over time in either systolic or diastolic blood pressure within the intervention group. An additional two studies also demonstrated a more modest, non-significant improvement in systolic blood pressure, diastolic blood pressure, or blood pressure control [31,32]. For the eight studies that reported difference-in-differences change in systolic blood pressure between the two study arms, there was a range of differences between study arms as large as 13.2 mmHg (P < 0.01) [27] and 12.45 mmHg (P < 0.05) [24] to as small as 0.37 mmHg (P = 0.43) [32], and six out of the eight studies reported statistically significant reductions in difference-in-differences change in systolic blood pressure.

**Table 3:** Overview of outcomes for intervention studies (compared to control if included).

Study	Mode of Communication	Intervention Type	Systolic Blood Pressure	Diastolic Blood Pressure	Percent Controlled Hypertension
Kingue et al., 2013 [26]	Telephone	Provider-provider consultation	++	++	++^a^
Liu et al., 2015 [24]	Telephone	Patient-provider behavioral counseling	++	++	
Kanadli et al., 2016 [25]	Telephone	Patient-provider behavioral counseling	++	+	
Sharma et al., 2017 [21]	Telephone	Patient-provider behavioral counseling	++	−	
Vitale et al., 2015 [22]	Telephone	Patient-provider medical management	+	++	
Rubinstein et al., 2016 [32]	Telephone	Patient-provider behavioral counseling	+	+^b^	
Patnaik et al., 2014^c^ [20]	Telephone	Patient-provider behavioral counseling	+/−^c^	+/−^c^	
Dange et al. 2019	Video chat	Patient-provider medical management	++	++	
Lee et al., 2018 [27]	Video chat	Patient-provider medical management	++	+	
Ruschel et al., 2020 [31]	Video chat	Provider-provider consultation			+
Nohara et al., 2015 [29]	Video chat	Patient-provider medical management	++		
Li et al., 2019 [23]	Electronic messaging	Provider-provider consultation	++	++	++
Nelissen et al., 2018 [28]	Electronic messaging	Provider-provider consultation	++	++	++
Patel et al., 2019 [30]	Electronic messaging	Provider-provider consultation	++	++	

Comparisons involving superior blood pressure changes are shaded blue, where (++) indicates superior with significance (P < 0.05) and (+) indicates superior without significance. Comparisons involving inferior blood pressure changes are shaded red, where (− −) indicates inferior with significance (P < 0.05) and (−) indicates inferior without significance. (+/−) indicates no difference.

^a^Subgroup of participants with stage I-II hypertension was inferior to control.

^b^Only for follow-up at 6 months.

^c^Reported as hypertension classification.

Of the nine studies that assessed patient-provider medical management or provider-provider consultation, five showed significant difference-in-differences changes and three showed significant pre-post changes in systolic or diastolic blood pressure. In contrast, only three out of the five studies that investigated interventions to modify behavioral risk factors resulted in significant difference-in-differences blood pressure reduction.

Of the seven studies that used telephone communication, five reported significant and one reported non-significant difference-in-differences improvements in outcomes related to systolic blood pressure, diastolic blood pressure, or blood pressure control. Out of the four studies that evaluated video chat applications, two demonstrated significant pre-post improvements and one demonstrated a significant difference-in-differences improvement in systolic blood pressure [19,27,29]. Of the three studies that analyzed electronic messaging platforms, two demonstrated significant difference-in-differences improvements and one showed significant pre-post improvement in both systolic and diastolic blood pressure [23,28,30].

#### Outcomes for other cardiometabolic conditions

In addition to outcomes related to blood pressure, several studies also reported additional cardiometabolic variables for diabetes and dyslipidemia: eight studies described outcomes related to diabetes and five studies reported outcomes for dyslipidemia (S3 Table). Out of the fourteen included studies, five demonstrated significant improvements in glycated hemoglobin or fasting blood glucose [19,21,22,24,25], and three showed significant reductions in total cholesterol [22,24,25]. In the studies showing significant improvements in blood glucose, the improvements in outcomes ranged from 26.5 mg/dL (P < 0.01) [19] to 1.6 mg/dL (P < 0.01) [21], and for the studies demonstrating significant improvements in total cholesterol, the outcomes ranged from 39 mg/dL (P < 0.001) [22] to 4.68 mg/dL (P < 0.05) [24].

## Discussion

Out of the fourteen unique studies included in this review, the majority (n = 11) demonstrated a significant improvement in either systolic or diastolic blood pressure, and two studies showed a non-significant improvement in either systolic blood pressure, diastolic blood pressure, or percentage of participants who achieved target blood pressure levels. Indeed, the broader literature on mobile health applications for NCD management in LMICs has generally shown promise for improving clinical outcomes, and our results contribute to the existing literature through demonstrating an overall positive impact of telemedicine for hypertension management [10–12,33].

Eight of the nine studies that investigated interventions to improve patient medical management displayed significant reductions in blood pressure, whereas only three of the five studies that assessed interventions to modify behavioral risk factors reported significant changes. Compared to medical management, interventions that promote lifestyle changes for patient self-management are typically multifaceted in nature and vary widely depending on patient-specific factors [33]. Nevertheless, our results suggest that telemedicine interventions that support the clinical management of hypertension may yield a larger reduction in blood pressure compared to those that only target lifestyle changes, potentially due to improved patient monitoring and treatment modification [34,35]. Our analysis also indicates that technologies that enable virtual consultation services can improve treatment approaches and significantly enhance blood pressure control measures. Thus, telemedicine technologies may be particularly useful in the setting of LMICs, where patients located in rural and underserved areas may have limited access to specialty consultation services [36].

Telephone was the most common mode of communication in the studies that showed a significant decrease in blood pressure, and telephone-based interventions comprised most of the reviewed studies. Interestingly, three of the four studies that incorporated video-chat and all three electronic messaging studies, all of which focused on medical management, demonstrated significant improvements in systolic or diastolic blood pressure. Depending on access to internet and online services, video chat or electronic messaging technologies may allow providers to interact more frequently with patients compared to telephone. However, our study does not have sufficient evidence to conclude that more frequent interaction is directly associated with greater blood pressure change. Prior literature reviews investigating the effects of frequency of telemedicine interventions on NCD management have been limited due to significant heterogeneity in reported studies, as seen in our review, as well as a lack of adequately powered, rigorous, and long-duration clinical trials [37,38]. However, the literature on self-monitoring of blood pressure has shown clinically significant improvements in blood pressure in combination with other co-interventions such as patient education or lifestyle counseling, which does correlate with frequency and intensity of the intervention [39].

Our scoping review has several limitations. We excluded studies that did not report results of interventions, were not peer-reviewed, or not written in English language, thereby excluding other potential technologies from consideration. The studies included in this review are heterogenous in nature with varying types of study designs, patient demographics, interventions, and reported outcomes. Thus, it is difficult to generalize the findings of this review to specific patient populations or to compare results across the studies given the lack of uniform study and intervention design. Our study is unable to provide insight into the overall effectiveness of the included interventions, as pooled analysis of the outcome data is not possible due to study heterogeneity. Further, most of the studies in our review involve multi-component interventions beyond telemedicine. Therefore, the impact of telemedicine on the reported outcomes cannot be definitively attributed to only the telemedicine component of the intervention, and future research will be necessary to elucidate how varying features of telemedicine interventions impact hypertension outcomes. Finally, as most of the included studies displayed improvements in blood pressure outcomes, our results may be subject to positive publication bias and thus underreport null or negative findings associated with telemedicine use.

Our results show that significant gaps still exist in the literature on telemedicine interventions for hypertension control in LMICs. Our review demonstrates that few studies have been conducted in lower-income countries, as eleven of the fourteen studies were performed in middle-income countries, which is consistent with previous research on mobile health interventions for NCD management in LMICs [12]. Furthermore, several studies included small sample sizes, short duration of follow-up, and heterogeneity of interventions. None of the studies included in our review performed a cost-effectiveness analysis or estimated the health system costs associated with the telemedicine interventions. Additionally, as telemedicine continues to become integrated into standard clinical practice, further research concerning patient and provider experiences, barriers to using telemedicine, and data security and regulatory considerations will also be needed to ensure long-term uptake and assess quality of care.

Our review demonstrates that the majority of fourteen LMIC studies assessing the impact of telemedicine technologies for hypertension management resulted in significant improvements in blood pressure outcomes. While our review offers promise for telemedicine as a strategy to improve access to care in resource-limited settings, definitive conclusions regarding the effectiveness, cost-effectiveness, and sustainability of telemedicine for hypertension is not currently possible. There remain significant gaps in the available literature for determining the overall efficacy of telemedicine interventions in LMICs as the overall number of relevant published studies of telemedicine for hypertension control in LMICs remains limited and narrow in scope. Our findings are also consistent with previous reviews of telemedicine interventions for hypertension control in high-income countries, which have also demonstrated an overall summary estimate supporting improvement in clinical blood pressure control but at the same time a significant degree of study heterogeneity in terms of design and outcomes [40]. In light of the COVID-19 pandemic, the importance of cost-effective approaches for the management of hypertension in resource-limited LMIC settings has become increasingly apparent. As current evidence suggests that telemedicine may be a promising approach to increase access to care and improve outcomes for hypertension in LMICs, governments can consider laying the regulatory, infrastructural, and operational groundwork now to facilitate introduction of telemedicine programs. Additional high-quality trials of sufficient size and duration are also needed to determine the overall efficacy of telemedicine interventions and establish telemedicine’s role for hypertension management in LMICs.

## Supporting information

S1 Table Search strategy for PubMed search on May 21, 2020. (DOCX)

S2 Table Overview of non-telemedicine mobile health components for included interventions. (DOCX)

S3 Table Overview of telemedicine interventions for hypertension management in LMICs (including other cardiometabolic outcomes). (DOCX)

S1 Data Preferred Reporting Items for Systematic reviews and Meta-analyses extension for Scoping Reviews (PRISMA-ScR) checklist. (DOCX)
