# Supplementary material for: Telemedicine interventions for hypertension management in low- and middle-income countries: A scoping review
Source: PLoS One. 2021 Jul 9;16(7):e0254222. doi: 10.1371/journal.pone.0254222 (PMC8270399; doi:10.1371/journal.pone.0254222)
Supplement: S1 Table — (DOCX) [file pone.0254222.s001.docx]

| **Search** | **PubMed Query** |
| --- | --- |
| #1 | "Telemedicine"[Mesh] OR “telemedicine”[tiab] OR "telehealth"[Mesh] OR “telehealth”[tiab] OR "electronic health"[tiab] OR “ehealth”[tiab] OR “ehealth”[Mesh] OR “telephone monitoring”[tiab] OR “phone-based”[tiab] OR “mobile technology”[tiab] OR “mobile health”[tiab] OR “mhealth”[tiab] |
| #2 | "lower income country"[tiab] OR "lower income countries"[tiab] OR "middle income country"[tiab] OR "middle income countries"[tiab] OR "low-income country"[tiab] OR "low-income countries"[tiab] OR "middle-income country"[tiab] OR "middle-income countries"[tiab] OR "low-income"[tiab] OR "middle-income"[tiab] OR “LMIC”[tiab] OR "developing countr*"[tiab] OR “developing country”[Mesh] OR “Afghanistan”[tiab] OR “Angola”[tiab] OR “Albania”[tiab] OR “Argentina”[tiab] OR “Armenia”[tiab] OR “American Samoa”[tiab] OR “Azerbaijan”[tiab] OR “Burundi”[tiab] OR “Benin”[tiab] OR “Burkina Faso”[tiab] OR “Bangladesh”[tiab] OR “Bulgaria”[tiab] OR “Bosnia”[tiab] OR “Herzegovina”[tiab] OR “Belarus”[tiab] OR “Belize”[tiab] OR “Bolivia”[tiab] OR “Brazil”[tiab] OR “Bhutan”[tiab] OR “Botswana”[tiab] OR “Central African Republic”[tiab] OR “China”[tiab] OR “Cote d'Ivoire”[tiab] OR “Cambodia”[tiab] OR “Cameroon”[tiab] OR “Democratic Republic of the Congo”[tiab] OR “Republic of the Congo”[tiab] OR “Colombia”[tiab] OR “Comoros”[tiab] OR “Cabo Verde”[tiab] OR “Costa Rica”[tiab] OR “Cuba”[tiab] OR “Djibouti”[tiab] OR “Dominica”[tiab] OR “Dominican Republic”[tiab] OR “Algeria”[tiab] OR “Ecuador”[tiab] OR “Egypt”[tiab] OR “Eritrea”[tiab] OR “Ethiopia”[tiab] OR “Fiji”[tiab] OR “Micronesia”[tiab] OR “Gabon”[tiab] OR “Georgia”[tiab] OR “Ghana”[tiab] OR “Guinea”[tiab] OR “Gambia”[tiab] OR “Guinea-Bissau”[tiab] OR “Equatorial Guinea”[tiab] OR “Grenada”[tiab] OR “Guatemala”[tiab] OR “Guyana”[tiab] OR “Honduras”[tiab] OR “Haiti”[tiab] OR “Indonesia”[tiab] OR “India”[tiab] OR “Iran”[tiab] OR “Iraq”[tiab] OR “Jamaica”[tiab] OR “Jordan”[tiab] OR “Kazakhstan”[tiab] OR “Kenya”[tiab] OR “Kyrgyz Republic”[tiab] OR “Kiribati”[tiab] OR “Lao PDR”[tiab] OR “Lebanon”[tiab] OR “Liberia”[tiab] OR “Libya”[tiab] OR “St. Lucia”[tiab] OR “Sri Lanka”[tiab] OR “Lesotho”[tiab] OR “Morocco”[tiab] OR “Moldova”[tiab] OR “Madagascar”[tiab] OR “Maldives”[tiab] OR “Mexico”[tiab] OR “Marshall Islands”[tiab] OR “North Macedonia”[tiab] OR “Mali”[tiab] OR “Myanmar”[tiab] OR “Montenegro”[tiab] OR “Mongolia”[tiab] OR “Mozambique”[tiab] OR “Mauritania”[tiab] OR “Mauritius”[tiab] OR “Malawi”[tiab] OR “Malaysia”[tiab] OR “Namibia”[tiab] OR “Niger”[tiab] OR “Nigeria”[tiab] OR “Nicaragua”[tiab] OR “Nepal”[tiab] OR “Nauru”[tiab] OR “Pakistan”[tiab] OR “Peru”[tiab] OR “Philippines”[tiab] OR “Papua New Guinea”[tiab] OR “North Korea”[tiab] OR “Paraguay”[tiab] OR “West Bank”[tiab] OR “Gaza”[tiab] OR “Romania”[tiab] OR “Russia”[tiab] OR “Rwanda”[tiab] OR “Sudan”[tiab] OR “Senegal”[tiab] OR “Solomon Islands”[tiab] OR “Sierra Leone”[tiab] OR “El Salvador”[tiab] OR “Somalia”[tiab] OR “Serbia”[tiab] OR “South Sudan”[tiab] OR “Sao Tome”[tiab] OR “Principe”[tiab] OR “Suriname”[tiab] OR “Eswatini”[tiab] OR “Syrian Arab Republic”[tiab] OR “Chad”[tiab] OR “Togo”[tiab] OR “Thailand”[tiab] OR “Tajikistan”[tiab] OR “Turkmenistan”[tiab] OR “Timor-Leste”[tiab] OR “Tonga”[tiab] OR “Tunisia”[tiab] OR “Turkey”[tiab] OR “Tuvalu”[tiab] OR “Tanzania”[tiab] OR “Uganda”[tiab] OR “Ukraine”[tiab] OR “Uzbekistan”[tiab] OR “St. Vincent”[tiab] OR “Grenadines”[tiab] OR “Venezuela”[tiab] OR “Vietnam”[tiab] OR “Vanuatu”[tiab] OR “Samoa”[tiab] OR “Kosovo”[tiab] OR “Yemen”[tiab] OR “South Africa”[tiab] OR “Zambia”[tiab] OR “Zimbabwe”[tiab] |
| #3 | "Hypertension"[Mesh] OR “hypertension”[tiab] OR “hyperten*”[tiab] OR “hypertensive”[tiab] OR “blood pressure”[Mesh] OR “blood pressure”[tiab] OR “high blood pressure”[tiab] OR “systolic blood pressure”[tiab] OR “diastolic blood pressure”[tiab] OR “raised blood pressure”[tiab] OR “elevated blood pressure”[tiab] OR “BP”[tiab] |
| #4 | #1 AND #2 AND #3 |
