# Supplementary material for: Telemedicine interventions for hypertension management in low- and middle-income countries: A scoping review
Source: PLoS One. 2021 Jul 9;16(7):e0254222. doi: 10.1371/journal.pone.0254222 (PMC8270399; doi:10.1371/journal.pone.0254222)
Supplement: S2 Table — (DOCX) [file pone.0254222.s002.docx]

| **Study** | **Non-telemedicine intervention** |
| --- | --- |
| Dandge et al. | Mobile health application that provided patients with appointment reminders and providers with access to patient electronic health records |
| Kanadli et al. | -- |
| Kingue et al. | Daily transmission of patient data via text messaging or voicemail to a central telemedicine center, which enabled providers to receive real-time feedback for treatment planning |
| Lee et al. | Patients provided with a blood pressure monitoring device to self-measure blood pressure as well as a mobile health application to input their blood pressure measurements for remote monitoring |
| Li et al. | Health education and health promotion materials sent to patients via WeChat, and patients self-reported blood pressure over WeChat to receive real-time feedback from researchers |
| Liu et al. | Computerized cardiovascular risk evaluations and treatment interventions as well as text messages for risk factor modification through patient mobile phones |
| Nelissen et al. | Mobile health application for remote patient monitoring of blood pressure values |
| Nohara et al. | Portable health clinic with sensor devices, a data transmission system, and a data management application |
| Patel et al. | Mobile application with shared electronic record capabilities and reminders for medication adherence; notification of high-risk individuals via text message or interactive voice response to attend follow-up appointments |
| Patnaik et al. | Text messaging to reinforce lifestyle and behavioral risk factor modification |
| Rubinstein et al. | Monthly phone calls and tailored text messaging to reinforce lifestyle and behavioral risk factor modification |
| Ruschel et al. | -- |
| Sharma et al. | Text messaging to reinforce lifestyle and behavioral risk factor modification |
| Vitale et al. | Patient-reported blood glucose values sent via phone, email, or secure website were evaluated by research team |
