# Supplementary material for: Telemedicine interventions for hypertension management in low- and middle-income countries: A scoping review
Source: PLoS One. 2021 Jul 9;16(7):e0254222. doi: 10.1371/journal.pone.0254222 (PMC8270399; doi:10.1371/journal.pone.0254222)
Supplement: S3 Table — (DOCX) [file pone.0254222.s003.docx]

| **Study (country)** | **Non-communicable disease(s)** | **Inclusion criteria** | **Sample size** | **Study design and follow-up** | **Description of telemedicine component of intervention** | **Outcomes (reported as difference in differences unless otherwise specified)** |
| --- | --- | --- | --- | --- | --- | --- |
| Dandge et al., 2019 (India) | Hypertension, diabetes | - Adults aged 20 years and above - Located in two randomly selected villages from Medchal | 1,835 (413 with hypertension, 189 with diabetes) | Cross-sectional with pre- and post-test analysis; follow-up after 24 months | - Video Skype call between the participant and the physician using mHealth application coordinated by non-physician health workers. | - 54% achieved BP control status - SBP ↓ 6.5 mmHg (P<0.001) and DBP ↓ 5.9 mmHg (P<0.001) among participants with a past medical history of hypertension - SBP ↓ 18.1 mm Hg (P<0.001) and DBP ↓ 15.3 mm Hg (P<0.001) among participants with newly detected hypertension - Fasting blood sugar ↑ 6.2 mg/dL (P=0.30) and HbA1c ↓ 0.3% (P=0.03) among participants with a past medical history of diabetes - Fasting blood sugar ↓ 26.5 mg/dL (P=0.007) and HbA1c ↓ 0.9% (P = 0.006) among newly detected participants with diabetes |
| Kingue et al., 2013 (Cameroon) | Hypertension | - Adults aged 15 years and above - SBP≥140 mmHg or DBP ≥90 mmHg - SBP≥130 mmHg or DBP≥80 mmHg for those with diabetes or nephropathy - At least 12 months of continuous residence in the study areas prior to the study | 268 (intervention = 165, control = 103) | Prospective interventional study; follow-up after 24 weeks | - Staff located at remote treatment centers could immediately consult with a hospital telemedicine center via mobile phone for clinical decision making. | - SBP ↓ (P=0.01) - DBP ↓ (P=0.0002) - 50% of intervention group and 39.1% of control group achieved target BP levels (P = 0.04) among participants with stage 3 hypertension - 65.2% of intervention group and 70% of control group achieved target BP levels (P = 0.20) among participants with hypertension stage I-II |
| Kanadli et al., 2016 (Turkey) | Diabetes | - Diagnosed with diabetes in the previous year | 88 (intervention = 44, control = 44) | Randomized controlled trial; follow-up after 3 months | - Received telephone follow-up weekly in the first month and biweekly in the second and third months. - Metabolic control values reviewed, and continued education provided during calls. | - SBP ↓ 5.91 mmHg (P=0.011) - DBP ↓ 2.72 mmHg (P=0.21) - HbA1c ↓ 0.35% (P<0.001) - Total cholesterol ↓ 17.05 mg/dL (P=0.035) - HDL ↓ 0.97 mg/dL (P=0.53) - LDL ↓ 6.89 mg/dL (P=0.19) |
| Lee et al., 2018 (Vietnam) | Hypertension, diabetes | - Overseas Korean patients with hypertension, diabetes mellitus, or both | 234 total; 36 received two or more telehealth counselling sessions (intervention = 10, control = 26) | Cross-sectional with pre- and post-test analysis; follow-up after 3 months | - Received telehealth counseling from a Korean doctor through a telehealth network system. | - SBP ↓ 6.4 mmHg and DBP ↓ 4.4 mmHg for all patients - SBP ↓ 13.2 mmHg (P=0.0076) between monitoring and non-monitoring group - DBP ↓ 1.7 mmHg (P=0.67) between monitoring and non-monitoring group |
| Li et al., 2019 (China) | Cardiovascular disease | - Adults aged 45-70 years old - Able to use WeChat - Lived in Yuexiu District for at least six months - Reported a definite diagnosis of hypertension while taking or having ever taken an antihypertensive drug | 462 (intervention = 186, control = 276) | Randomized controlled trial; follow-up after 6 months | - Private chats between participants and researchers via WeChat. - Researchers consulted with community physicians for hypertension management plans via WeChat. | - SBP ↓ 6.9 mmHg (P=0.002) - DBP ↓ 3.1 mmHg (P=0.016) - Hypertension control ↑ 22.7% (adjusted odds ratio: 5.0 (2.3, 11.3); P < 0.001) in the intervention group |
| Liu et al., 2015 (China) | Cardiovascular disease | - Adults aged 45-75 years without known cardiovascular disease | 589 (intervention = 238, control = 351) | Randomized controlled trial; follow-up after 12 months | - Received phone calls ranging from once per week to twice per month depending on 10-year risk of CVD. - Calls focused on providing guidance related to modifiable risk factors and healthy lifestyle. | - SBP ↓ 12.45 mmHg (P<0.05) - DBP ↓ 12.23 mmHg (P<0.01) - Fasting plasma glucose ↓ 0.32 mmol/L (P < 0.001) - Total cholesterol ↓ 0.26 mmol/L (P<0.05) - HDL ↑ 0.03 mmol/L (P>0.05) - LDL ↑ 0.03 mmol/L (P>0.05) |
| Nelissen et al., 2018 (Nigeria) | Hypertension | - Adults aged 18 years and above - New or previous hypertension diagnosis | 336 | Cross-sectional with pre- and post-test analysis; follow-up after 6 months | - Pharmacists communicated with the cardiologists via an mHealth app for remote patient management. | - SBP ↓ 9.9 mmHg (P<0.05) - DBP ↓ 5.4 mmHg (P<0.05) - BP on target ↑ 32% (P < 0.001) |
| Nohara et al., 2015 (Bangladesh) | Hypertension, diabetes | - Adults from rural and urban areas in Bangladesh | 16,741 (2 361 participated in both health checkups) | Cross-sectional with pre- and post-test analysis; follow-up after 12 months | - Patients classified as either "affected" or "emergent" were provided telemedicine consultations via Skype with a medical call center. | - Identified 32.4% of subjects as affected and 54.1% of subjects as caution required - Mean SBP ↓ 5 mmHg for all participants after second health checkup (P<0.001) |
| Patel et al., 2019 (Indonesia) | Cardiovascular disease | - Adults aged 40 years and older - Living in one of eight intervention or control villages | 22,635 (intervention = 11 647, control = 10 988) | Randomized controlled trial; follow-up after 11.5 (intervention initiation to end of follow up), 12.6 months (control) | - Physicians and nurses received tailored decision support for treatment plans via a mobile application - Treatment plans subsequently sent to community health workers. | - SBP ↓ 8.3 mmHg (P<0.001) - DBP ↓ 3.6 mmHg (P<0.001) |
| Patnaik et al., 2014 (India) | Diabetes mellitus, coronary heart disease | - Adults aged 30 years and above - Treated for diabetes for at least 3 months | 100 (intervention = 50, control = 50) | Randomized controlled trial; follow-up after 3 months | - Participants contacted every 3 weeks for 3 months by telephone by the investigator, - Asked about lifestyle changes and provided counseling. | - No significant change in hypertension classification between intervention and control groups - Postprandial blood sugar ↑ 5 mg/dL (no significance reported) - Fasting blood sugar ↑ 4.3 mg/dL (no significance reported) - Serum cholesterol ↓ 2 mg/dL (no significance reported) |
| Rubinstein et al., 2016 (Argentina, Guatemala, Peru) | Hypertension | - Adults 30-60 years old who owned mobile phones - SBP and DBP in the prehypertension range (between 120 and 139 mmHg and between 80 and 89 mm Hg, respectively) - Not receiving medication for hypertension | 637 (intervention = 316, control = 321) | Randomized controlled trial; follow-up after 12 months | - Monthly phone calls using motivational interview techniques. - Discussed lifestyle modifications (e.g., reduction of sodium intake, promotion of physical activity). | - SBP ↓ 1.13 mmHg (P=0.31) at 6 months and ↓ 0.37 mmHg (P=0.43) at 12 months - DBP ↓ 0.45 mmHg (P=0.44) at 6 months and ↑ 0.01 mmHg (P=0.99) at 12 months |
| Ruschel et al., 2020 (Brazil) | Coronary artery disease | - Adults aged 18 years or older - Diagnosed with coronary artery disease and class I or II angina meeting discharge criteria - No cardiovascular event or decompensated clinical condition in past year | 271 (intervention = 135, control = 136) | Randomized controlled trial; follow-up after 12 months | - Patients were followed-up after discharge from a specialized outpatient clinical to a primary care unit with clinical support from a cardiologist available for telemedicine consults. - Patients were interviewed via telephone. | - BP control ↑ 1.10% (P>0.05) - Controlled HbA1c (<7%) ↑ 15.56% (P>0.05) |
| Sharma et al., 2017 (India) | Non-communicable diseases (NCD) | - Adults aged 18 to 64 years who had been living in Barwala for at least 6 months | 400 (intervention = 200, control = 200) | Cross-sectional with pre- and post-test analysis; follow-up after 8 months | - Patients received telephone calls once a month and discussed behavioral modification of NCD risk factors and any questions with the researchers. | - SBP ↓ 1.3 mmHg in intervention group (P<0.001) and ↑ 0.3 mmHg in control group (P=0.08) - DBP ↑ 0.3 mmHg in intervention group (P=0.59) and ↑ 0.1 mmHg in control group (P=0.47) - Fasting blood glucose ↓ 1.4 mg/dL in the intervention group (P=0.006) and ↑ 0.2 mg/dL in the control group (P=0.10) - Total cholesterol ↓ 0.2 mg/dL in the intervention group (P=0.46) and ↓ 0.3 mg/dL in the control group (P=0.07) - HDL ↑ 0.2 mg/dL in the intervention group (P=0.010) and ↓ 0.1 mg/dL in the control group (P=0.32) - LDL ↓ 0.5 mg/dL in the intervention group (P=0.28) and ↑ 0.1 mg/dL in the control group (P=0.07) |
| Vitale et al., 2015 (India) | Diabetes | - Adults between 25 and 80 years old - Diagnosed with diabetes more than a year previously | 175 (intervention = 100, control = 75) | Cross-sectional with pre- and post-test analysis; follow-up not reported | - Patients provided with telephone appointments with a multi-disciplinary team of diabetologists, diabetes educators, dietitians, pharmacists, and psychologists. | - SBP ↓ 4.1 mmHg (P=0.20) - DBP ↓ 4.1 mmHg (P=0.016) - HbA1c ↓ 0.96% (P=0.003) - Total cholesterol ↓ 39 mg/dL (P<0.001) |

BP, blood pressure; SBP, systolic blood pressure; DBP, diastolic blood pressure; HDL, high-density lipoprotein; LDL, low-density lipoprotein; HbA1c, hemoglobin A1c
